# Blood Cell Membrane-Coated Nanomaterials as a Versatile Biomimetic Nanoplatform for Antitumor Applications

**DOI:** 10.3390/nano14211757

**Published:** 2024-10-31

**Authors:** Hanchun Shen, Yongliang Ouyang, Liang Zhang, Jing Li, Shige Wang

**Affiliations:** School of Materials and Chemistry, University of Shanghai for Science and Technology, Shanghai 200093, China; 222143044@st.usst.edu.cn (H.S.); 222412781@st.usst.edu.cn (L.Z.)

**Keywords:** nanomaterials, blood cells, blood cells membrane, tumor therapy

## Abstract

The application of nanomaterials in tumor therapy is increasingly widespread, offering more possibilities for enhanced tumor therapy. However, the unclear biological distribution and metabolism of nanomaterials may lead to immune rejection or inflammatory reactions, posing numerous challenges to their clinical translation. The rich diversity and multifaceted functions of blood cells offer promising biological avenues for enhancing the application of nanoparticles in cancer therapy. Blood cell membranes, being made of naturally found components in the body, exhibit significant biocompatibility, which can reduce the body’s immune rejection response, extend the drug’s residence time in the bloodstream, and enhance its bioavailability. Integrating blood cell membranes with nanomaterials enhances tumor therapy by improving targeted delivery, prolonging circulation time, and evading immune responses. This review summarizes recent advancements in the application of blood cell membrane-coated nanomaterials for antitumor therapy, with a particular focus on their use in photodynamic and photothermal treatments. Additionally, it explores their potential for synergistic effects when combined with other therapeutic modalities.

## 1. Introduction

Modern medical technology has made significant advances in the detection, prevention, and treatment of malignant tumors. However, clinical statistics indicate that the annual number of cases of malignant tumors is still growing [1]. Traditional cancer treatment methods are surgery, radiation therapy, and chemotherapy. Unfortunately, surgical treatment is prone to complications, and both chemotherapy and radiotherapy can cause damage to normal cells [2]. Meanwhile, novel treatment protocols such as gene editing and virus- and nanoparticle-mediated cancer therapy have been proposed [3]. The combination of nanomaterials with conventional treatment methods can better reduce the side effects of the tumor therapy process. The effects of nanoparticles as a drug delivery system have been extensively documented in clinical and experimental studies over the past decades. Photothermal therapy (PTT) of tumors utilizes photothermal conversion agents to absorb light energy and convert it into heat to destroy tumor cells. Photodynamic therapy (PDT) of tumors is a method that uses photosensitizers and specific wavelengths of laser to kill tumor cells [4,5]. Nanomaterials have been widely applied in PTT and PDT for tumors. The main challenges and shortcomings in nanoparticle-mediated tumor PTT and PDT include easy clearance by the immune system, a very short blood half-life, and low enrichment at the tumor site. Moreover, nanomaterials are usually injected into the human body via intravenous injection, and blood cells will first contact with the active ingredients of the nanomedicine. Therefore, improving biocompatibility and reducing cytotoxicity have also become one of the primary issues in the development of nanomedicines for tumor PTT and PDT [6]. In a classic approach, polyethylene glycol (PEG) has been complexed with various nanocarriers to prevent recognition by the endogenous mononuclear phagocytic system and enable long-term circulation in vivo [7]. PEG acts as a hydrophilic protective layer and can further improve the targeted delivery of drugs [8,9]. However, the PEG modification of nanoparticles exhibits low efficiency. To address this issue, nanomaterials have been coated with blood cell membranes to create cell membrane-based biomimetic nanomaterials.

Recent advances in biomedical technology have greatly enhanced the tumor-targeting capability and multifunctionality of nanomaterials. However, during practical applications, proteins within the human body tend to adsorb onto the surface of nanomaterials, forming a protein corona, which hinders their targeted delivery efficacy [10,11,12]. To overcome these challenges, bio-inspired strategies have been proposed to integrate biomembranes, organelles, peptides, and other cellular components onto nanomaterials [13,14,15]. This approach aims to overcome biological barriers, target diseased tissues, and mitigate immune responses, thereby further optimizing the biomimetic properties of nanoparticles [16,17]. Membranes of macrophages, neutrophils, and red blood cells (RBCs) are easy to prepare and collect. Coated nanoparticles with membranes such as red cell membrane, white cell membrane, and platelet membrane can minimize the nanoparticles cytotoxicity. As host substances, these membranes can avoid the immune rejection. In addition, these biomimetic nanomaterials can achieve an efficient targeted delivery by imitating the composition and action mechanism of some cells in the body [18]. In some cases, nanoparticles coated with a membrane can enhance inflammatory cytokine secretion, immune cell maturation, and the targeting of lymph node-resident dendritic cells [19]. The coating of a neutrophil membrane can further render the nanomaterials with chemotaxis to target the inflammatory sites [20].

**Scheme 1:**
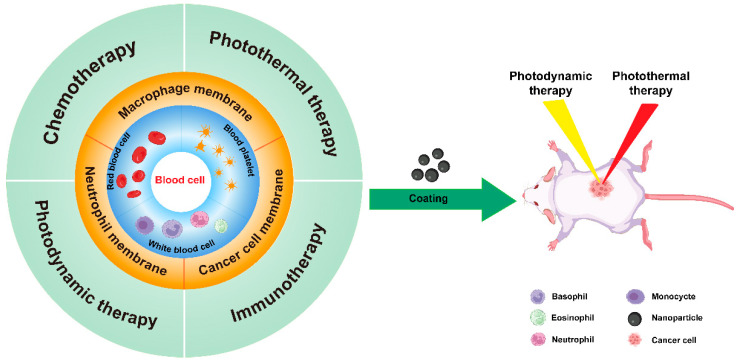
This schematic illustrates the application of nanomaterials in combination with various types of blood cell membranes for tumor therapy, emphasizing the utilization of blood cell membrane-encapsulated nanomaterials in PDT and PTT.

The unique properties of blood cells, including their source and characteristics, offer solutions for enhancing the targeted delivery and biocompatibility of nanomaterials, thus increasing their versatility. This review focuses on the role of blood cell membranes in conjunction with nanomaterials for the treatment of PDT and PTT. Additionally, we provide a concise overview of the involvement of blood cells in combination therapies, including immunotherapies and chemotherapies (Scheme 1). This discussion aims to deepen our understanding of the critical functions of blood cell membranes in oncological treatment strategies.

## 2. Blood Cells as Vehicles for Nanoparticle Delivery Systems

### 2.1. Red Blood Cells in Nanoparticle Delivery Systems

Blood cells, as natural cellular entities, represent one of the most accessible raw materials. Among them, RBCs, white blood cells, and platelets are classified as blood cells. RBCs, constituting approximately 99% of all blood cells, are easily separable from donor blood, making them an ideal source of natural cell membranes [21,22]. Physiologically, RBCs possess a biconcave discoid shape that provides a substantial surface area. With sizes ranging between 7–8 μm, and lacking a nucleus and mitochondria, they offer ample internal volume for loading and encapsulating nanoparticles. The encapsulation of nanoparticles within RBC membranes endows them with self-recognition, high biocompatibility, and an extended circulation lifespan in blood [23]. Typically, RBC membranes can be isolated through the centrifugation of whole blood at 4 °C, a process designed to preserve protein activity while effectively retrieving RBCs. Subsequently, the centrifuged products undergo washing and purification using a phosphate buffer solution. Finally, the RBCs are subjected to hypotonic lysis, facilitated by ultrasonic fragmentation, to release intracellular components and obtain the desired RBC membranes [24,25]. Li et al. bound 1,2-distearoyl-sn-glycero-3-phosphoethanolamine-N-[folic acid (polyethylene glycol)-2000] (DSPE-PEG-FA) molecules to the RBC membrane, which was further reconstituted into vesicles and encapsulated with upconversion nanoparticles (UCNPs). The resulting final material, FA-RBC-UCNPs, acquired excellent stealth properties from the RBC source, helping the material to avoid immune attack and also prolonging blood retention time [26]. In another study, Zhang and colleagues designed an antigenic peptide delivery system with RBC membrane-encapsulated poly D, L-lactic-co-glycolicacid nanoparticles (PLGA-NPs) [27]. The RBC membrane was modified by incorporating distearoyl phosphatidylethanolamine-polyethylene glycol-mannose (DSPE-PEG-mannose), resulting in DSPE-PEG-Man-inserted RBCs. This modification facilitates the specific targeting of antigen-presenting cells within lymphoid organs, while simultaneously protecting the antigen from systemic clearance and extending the circulation time of the antigen carriers encapsulated within the nanoparticles.

### 2.2. Platelets and White Blood Cells

Apart from red blood cells, other blood cells such as platelets and white blood cells possess specific surface receptors [17,28,29]. These receptors (GPIIb/IIIa and P-selectin) can selectively bind to activated endothelial cells and tumor cells within the tumor microenvironment [30,31]. This interaction enables platelets to selectively aggregate at tumor sites, thereby facilitating direct delivery of therapeutics to malignant tissues. Leveraging this selectivity, Rao et al. harvested platelet-derived vesicles/membrane proteins from murine blood, which were enveloped around Fe_3_O_4_ magnetic nanoparticles (MNs) to create the integrated nano-platform known as PLT-MNs [32]. Compared with RBC-MNs wrapped with red blood cell membranes, PLT-MNs have both long-circulation targeting ability and enhanced tumor MRI.

White blood cells play a pivotal role in the formation of these physiological barriers [33,34]. During inflammation or infection, they migrate to these areas, clearing pathogens and damaged cells, releasing cytokines, and regulating local immune responses. Due to their ability to synergize with immune cells, white blood cells prevent rapid clearance of drugs by phagocytic cells within the immune system. Additionally, through receptor-ligand interactions, they facilitate communication with endothelial cells, thereby reconstructing endothelial transport during inflammation and facilitating payload release. White blood cells are further classified into granulocytes and lymphocytes. Chen and colleagues utilized endogenous neutrophils as natural drug carriers, integrating AgAuSe quantum dots loaded onto peptide–polysaccharide multilayer bacterial networks. This innovative approach successfully achieved integrated NIR-II imaging and sonodynamic therapy [35]. It is noteworthy that the membrane preparation process for eukaryotic cells, such as leukocytes, differs from that of anucleated cells like red blood cells and platelets. It typically necessitates the disruption of the cell membrane via ultrasound, followed by centrifugation to eliminate soluble proteins. Subsequently, core NPs and the extracted membrane vesicles are fused through techniques such as ultrasonication, electroporation, and extrusion, facilitating the assembly of the core-shell structure [36,37,38,39].

## 3. Application of Blood Cell Membrane-Coated Nanomaterials in PTT of Tumors

### 3.1. Advancements in RBC Membrane-Coated Nanomaterials for Enhanced PTT

**Figure 1:**
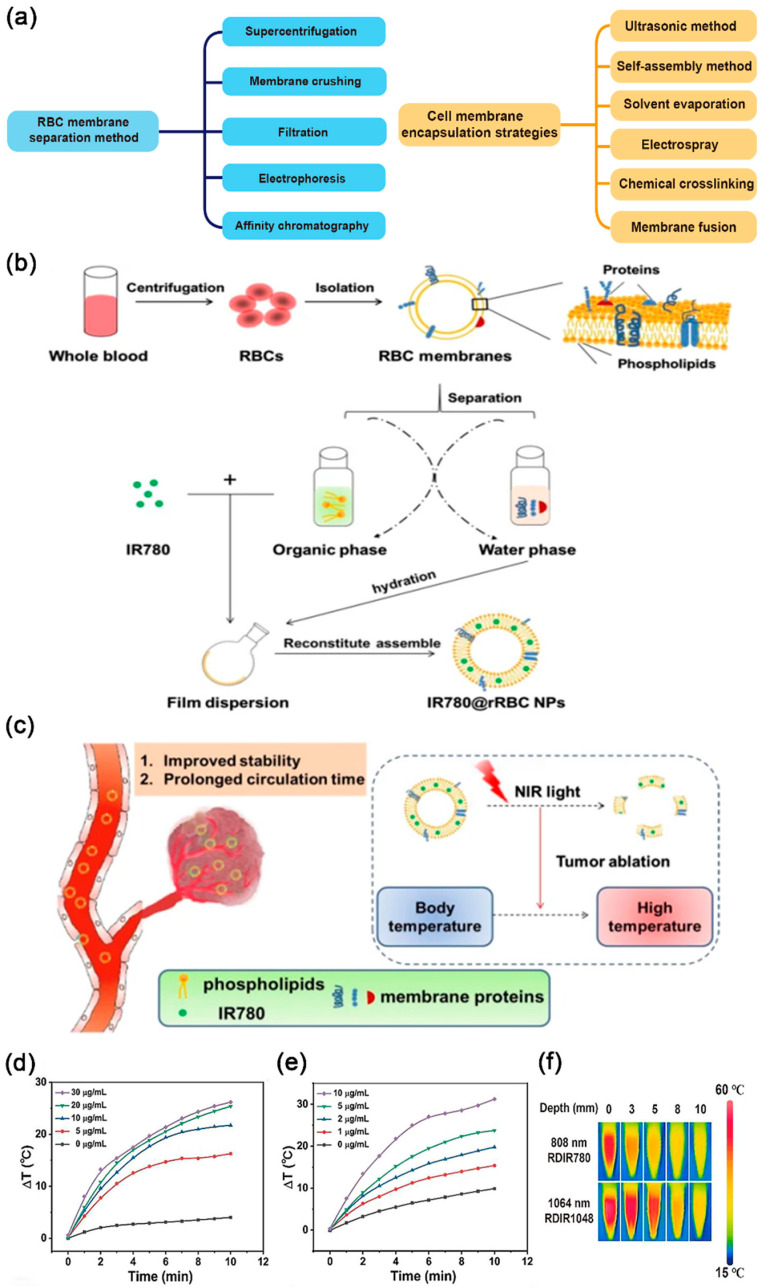
(**a**) A concise overview of the techniques for RBC membrane separation and the strategies associated with the encapsulation of nanomaterials. (**b**) The process of preparing IR780@rRBC NPs. (**c**) IR780@rRBC schematic diagram of the in vitro stability and circulatory capacity of NPs. (**b**,**c**) Reprinted with permission from ref. [53]. Copyright Springer Publishers. (**d**) Photothermal heating profiles were obtained using different concentrations of DIR780. (**e**) Photothermal heating profiles were obtained using different concentrations of DIR1048. (**f**) In vitro thermograms of DIR780 and DIR1048. (**d**–**f**) Reprinted with permission from ref. [54]. Copyright American Chemical Society publishers.

During PTT, photothermal conversion agents absorb energy from photons and transform from their ground singlet state to an excited singlet state. Subsequently, the photothermal conversion agents undergo a vibrational relaxation process, returning from the excited state to the ground state, and the energy generated during this process heats the microenvironment around the photothermal agent [40]. This process destroys tumor cells and harmful tissue cells via hyperthermia. Many photothermal agents have been applied in clinical practice, such as carbon nanomaterials, gold nanomaterials, silver nanomaterials, and germanium nanocrystals [41,42,43,44]. These exogenous nanoparticles (NPs) can be coated with endogenous cell membranes like RBC, platelet, leukocyte, cancer cell, and hepatocyte membranes. Extracting membranes from eukaryotic cells, such as leukocytes, cancer cells, and hepatocytes, poses challenges due to its complexity. Conversely, utilizing blood cells to coat diverse nanomaterials, such as liposomes, silica, mesoporous silica, gold particles, and metal-organic frameworks, is easy [45,46,47]. This is because blood cells can be easily isolated from blood donors. Moreover, mature RBCs lack a nucleus and organelles; most of the cell’s interior can be utilized for drug loading and can undergo complete degradation without producing toxic metabolites [48]. Several methods are available for the separation and extraction of RBC membranes, including ultracentrifugation, membrane fragmentation, and electrophoresis (Figure 1a) [49,50]. The encapsulation techniques for the corresponding NPs primarily encompass ultrasonic methods, self-assembly approaches, and chemical cross-linking methods [51].

You et al. coated gold nanocages with RBC membrane, demonstrating the excellent photothermal effect in vitro under NIR laser irradiation. In the 4T1 mouse tumor model, the PTT of the RBC membrane-gold nanoparticle group exhibited effective tumor ablation, with a significantly faster decrease in average tumor volume compared to the control group. This can be attributed to the two-fold increase in tumor uptake rate after RBC coating, along with longer retention detected in other organs [52]. In another study, Wang et al. isolated endogenous proteins and lipids from natural RBC membranes and bound them with IR780 through membrane dispersion to investigate their potential impact on PTT performance (Figure 1b,c) [53]. In the tumor photothermal treatment on mice, it was found that the temperature of the IR780@RBC NPs and the NPs prepared from biomimetic recombinant RBC membranes (IR780@rRBC NPs) under near-infrared irradiation was 10 °C higher than that of the IR780 alone. After laser irradiation, both IR780@RBC and IR780@rRBC NPs were weaker in cytotoxicity than free IR780, but both cellular uptakes showed favorable results and easier access to the treatment site. In addition, free IR780, IR780@RBC, and IR780@rRBC NPs were subjected to in vivo pharmacokinetic studies, and the half-life in plasma of IR780@rRBC was also significantly prolonged compared to free IR780 or IR780@RBC.

**Figure 2:**
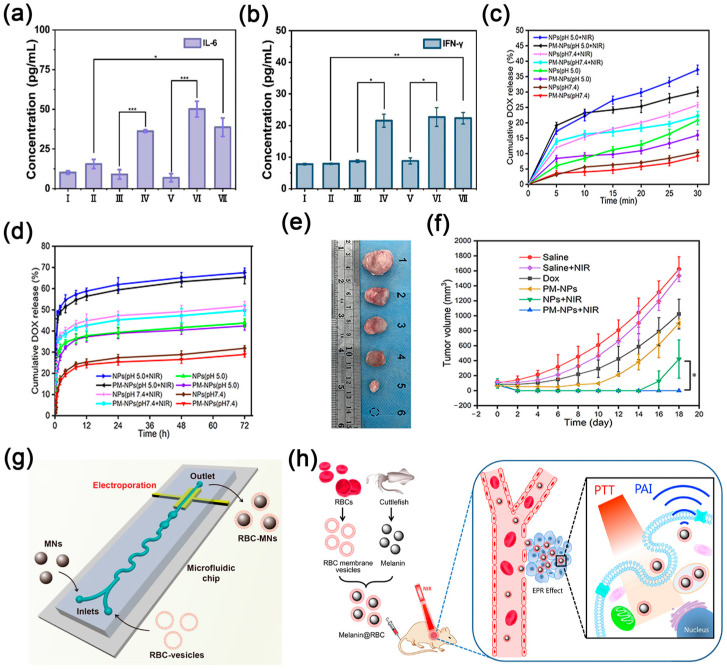
(**a**) IL-6, and (**b**) IFN-γ in the cell supernatant after different treatments using the ATP assay kit and enzyme-linked immunosorbent assay. Group information: I. PBS, II. anti-PD-L1 antibody, III. DIR1048, IV. DIR1048 + laser, V. RDIR1048, VI. RDIR1048 + laser, and VII. RDIR1048 + anti-PD-L1 antibody + laser. *: *p* < 0.05, **: *p* < 0.01, ***: *p* < 0.001 (**a**,**b**) Reprinted with permission from ref. [54]. Copyright American Chemical Society publishers. After (**c**) 30 min and (**d**) 72 h, cumulative DOX release from NPs and PM-NPs at pH 5.0 or 7.4 with or without laser irradiation. (**e**) Representative images of the tumors after different treatments on day 18. (**f**) Tumor growth in tumor-bearing mice by treatment groups. Group information: (1) saline, (2) saline + laser, (3) Dox, (4) PM-NPs only. (**c**–**f**) Reprinted with permission from ref. [55]. Copyright Dove Medical publishers. (**g**) Microfluidic electroporation-facilitated synthesis of RBC-MNs for enhanced imaging-guided cancer therapy. Reprinted with permission from ref. [57]. Copyright American Chemical Society publishers. (**h**) Schematic illustration of RBC membrane-coated melanin nanoparticles for enhanced PTT. Reprinted with permission from ref. [58]. Copyright Elsevier publishers.

In a recent study, Yin and co-workers used nanoprecipitation and microemulsion techniques to mix distearoyl phosphatidylethanolamine-modified PEG with NIR dyes IR1048 and IR780, respectively [54]. The resulting NPs were designated as DSPE-PEG-OH@IR780 (DIR780) and DSPE-PEG-OH@IR1048 (DIR1048), respectively. Both DIR780 and DIR1048 were subsequently encapsulated with RBC membranes. Under laser irradiation at 808 nm for 10 min with a fixed NP concentration of 10 mg/mL, both DIR780 and DIR1048 demonstrated photothermal conversion efficiencies exceeding 25%. Notably, DIR1048 achieved a remarkable photothermal conversion efficiency of 43.2% (Figure 1d–f). After 24 h of co-incubation with differentially treated 4T1 cells, both IL-6 and IFN-γ levels in the dendritic supernatants of the DIR780 and DIR1048 groups increased. This elevation indicates that PTT can induce immunogenic cell death (ICD), thereby activating dendritic cells and enhancing the antitumor efficacy (Figure 2a,b). Additionally, the utilization of RBC membranes for DIR1048 facilitates immune evasion, resulting in prolonged in vivo retention. This decreased sequestration by the reticuloendothelial system (RES) promotes increased accumulation of DIR1048 at tumor sites.

### 3.2. Innovative RBC and NPs Encapsulation Techniques

Exploring novel modifications of RBC-coated NPs further promotes the PTT and PDT of tumors. Niu et al. employed IR780 and DOX as photothermal agents and antitumor drugs [55]. They combined these with polylactic-co-glycolic acid (PLGA) to prepare IR780@PLGA/DOX NPs. Subsequently, RBC membranes were used to coat the outermost layer, resulting in the formation of PM-NPs. After laser irradiation, the drug release from PM-NPs was significantly increased in both pH environments, and the cumulative release reached nearly 70% at 72 h. The drug release from PM-NPs was significantly accelerated in the acidic environment. The drug release profiles of the same materials under varying pH conditions demonstrate significantly higher values in acidic environments compared to alkaline conditions for NPs (Figure 2c,d). In the acidic environment, the drug release was significantly accelerated, but the release rate of PM-NPs was lower than that of IR780@PLGA/DOX NPs, indicating that the RBC membrane coating could lead to a smoother and longer-lasting drug release. Tumor images of hormonal mice demonstrated that the use of PM-NPs plus PTT can sufficiently ablate tumors with good oncological results (Figure 2e,f). In another study, Tuan et al. engineered a double-membrane hybrid using a combination of RBC and platelet membranes to coat polypyrrole nanoparticles (PPy NPs) [56]. First, the membrane-coated PPy NPs were injected into the mice through the tail vein. Under NIR irradiation, photothermal stimulation caused tumor blood vessels to break and form a large number of microthrombi. Moreover, the platelet membrane coating of PPy NP can target the injured tumor blood vessels and initiate a cascade that leads to clot formation and initiates the healing process. Subsequently, repeated laser irradiation induces an enhanced formation of microthrombi, leading to the accumulation of more PPy NPs in the compromised tumor blood vessels.

In the aforementioned studies, the preparation of blood cell membrane-coated NPs employs predominantly passive methods. Exploring new ways to encapsulate carriers more efficiently has also become one of the research priorities. Guo et al. proved that microfluidic electroporation can promote the entry of nanomaterials into the RBC vesicles so that the RBC membrane can more completely coat NPs, thus improving the PTT performance in vivo (Figure 2g) [57]. In another study, Liu and colleagues extracted natural melanin nanoparticles from living cuttlefish [58]. Then, they developed RBC membrane-coated melanin nanoparticles and exploited the photoacoustic imaging properties of the melanin nanoparticle core. The RBC membrane coating further promoted the immune escape ability of NPs and enhanced their accumulation in the tumor (Figure 2h). In vivo experiments on mice showed that the NPs have a good photothermal effect at low power density, and the anti-tumor rate was increased to almost 100%.

## 4. Application of Blood Cell Membrane-Coated Nanomaterials in PDT of Tumors

### 4.1. PSs and the Mechanism of PDT

PDT uses a certain light to stimulate photosensitizers (PSs) and produce ROS, which can damage cell structure and kill tumor cells. When a PS absorbs energy, its electrons move from the ground state (S_0_) to the excited singlet state (S_1_). These excited electrons then transition internally to the excited state of PS with the least vibrational energy within S_1_, swiftly undergoing relaxation [59]. In this process, the electrons in the PS-excited state return to the ground state through three processes, including fluorescence, vibration relaxation, and intersystem crossing. A non-radiative (intersystem crossing) transition is an equipartition vibrational order that converts an excited electron from S_1_ to a triplet state (T_1_) by a change in electron spin orientation. After the electrons vibrate and relax quickly in T_1_, PS molecules release energy in the form of phosphorescence and decay to the ground state. In the presence of molecular oxygen (^3^O_2_), the triplet state of the PS may relax by triggering photochemical reactions, driving ROS production, and causing damage to tumor cells [60].

ROS are highly active ions and free radicals, including superoxide (O^2−^), hydroxyl radical (·OH), hypochlorite ion (OCl^−^), hydrogen peroxide (H_2_O_2_), singlet oxygen (^1^O_2_), etc. [61,62]. In the presence of light, the triplet-state PS and organic or oxygen molecules in the cellular environment can generate ROS via processes of electron transfer, known as the type I mechanism. The type II mechanism involves the transfer of energy from the PS-excited triplet state to molecular oxygen, resulting in the generation of singlet oxygen (^1^O_2_). ROS molecules (like H_2_O_2_, ·OH, and ^1^O_2_) can easily pass through cell membranes, thus causing damage to cell structures and substructures [63]. Meeting the requirements of controlling ROS levels, targeting the tumor site, and minimizing the cytotoxic effects on normal cells can offer a more comprehensive therapeutic strategy for advancing the clinical study of tumor PDT.

The majority of photosensitizers feature a tetrapyrrole structure. An effective PS requires specific attributes: non-toxicity, hydrophilicity, suitability, sensitive photodynamic response, and activation via clinically utilized light wavelengths [64]. Lipson et al. demonstrated for the first time that porphyrin-type compounds have potential for the treatment of malignant tumors [65]. However, this PS underwent slow metabolism in normal cells, necessitating patients to shield themselves from bright light for several weeks post-treatment to prevent severe skin reactions [66,67]. Meanwhile, the drawbacks of the first generation of photosensitizers become evident: they exhibit an absorption peak at 630 nm, and the 630 nm light has a limited deep-tissue penetration. Moreover, they showed notable side effects, resulting in skin photosensitivity persisting for up to 6 weeks [68]. The second generation of photosensitizers features an expanded absorption spectrum, facilitating metabolic processes in the body and improving the efficiency of reactive oxygen species production. Porphyrin derivatives, metal phthalocyanine, and cycloquinone are among the components of the second generation of PSs. In addition, phthalocyanine PS has also attracted the attention of many researchers. It exhibits excellent long-wavelength absorption in the range of 650–800 nm and has an efficient singlet oxygen production capacity, which can destroy both shallow and deep tumors. However, the poor water solubility of phthalocyanine PS limits its application. Many methods have been developed to ameliorate this problem, such as conjugation with antibodies, proteins, and peptides [69,70,71,72]. In vivo studies using a monoclonal antibody linked to a tumor-specific antigen alongside zinc phthalocyanine indicate that larger antibody sizes hinder tissue permeability and decrease cellular uptake [73]. Moreover, attaching PSs to monoclonal antibodies could potentially diminish their antigen-specificity. The nano delivery system provides a new idea for improving the water dispersion and stability of a PS. The combination of nanocarriers and PS for PDT has the following advantages: (1) The large surface area can increase the load of PSs and improve its cellular uptake. (2) The nanoparticle carrier can protect a PS from premature release, thus reducing its concentration in normal tissues and inhibiting the overall phototoxicity. (3) Nanoparticles can induce amphiphilicity in a PS, facilitating its passage through the bloodstream and enabling targeted delivery to tumor tissue.

### 4.2. Blood Cell Membrane-Coated Nanomaterials in PDT for Tumors

**Figure 3:**
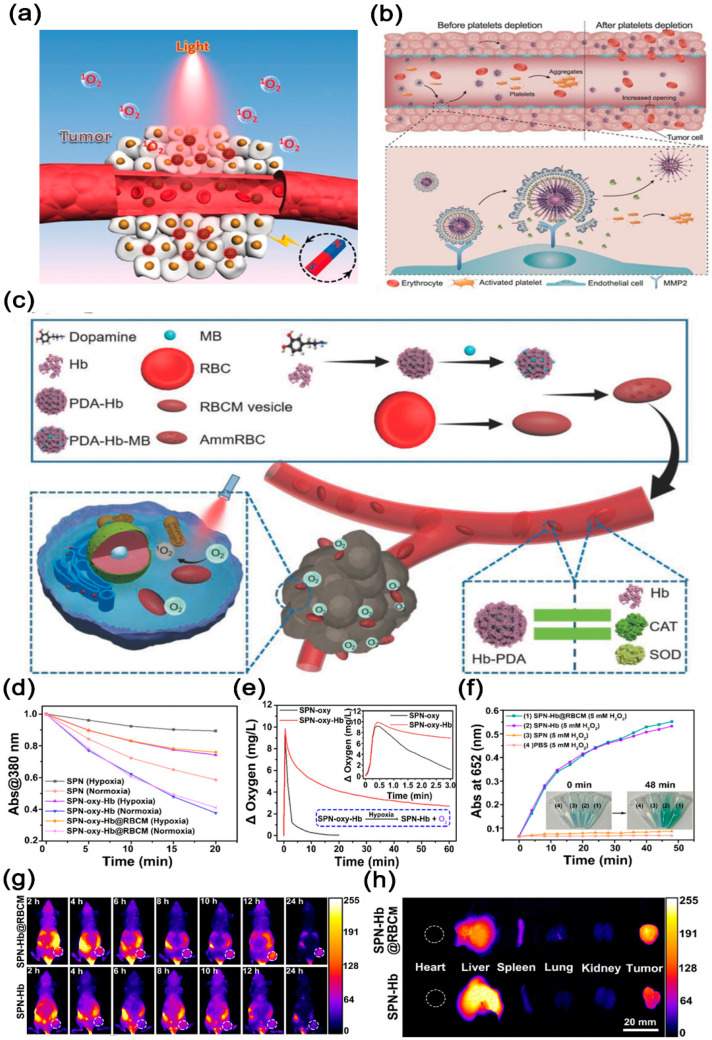
(**a**) The cloaking of magnetic nano-silica particles with natural RBC membrane was used in PDT. Reprinted with permission from ref. [74]. Copyright Wiley Publishers. (**b**) Lipid-polymer hybrid nanoparticles loaded with anti-platelet antibodies for selective depletion of intratumoral platelets. Reprinted with permission from ref. [75]. Copyright Oxford Univ Press publishers. (**c**) Schematic illustration of RBC membrane-camouflaged dimeric prodrug NPs (RBC(M(TPC-PTX))). Reprinted with permission from ref. [77]. Copyright American Chemical Society publishers. (**d**) After a period of laser irradiation (808 nm, 0.3 W/cm^2^), ^1^O_2_ generation was determined for SPN, SPN-oxy-Hb, and SPN-oxy-Hb@RBCM (normoxic or hypoxic conditions). (**e**) Oxygen release profiles of oxygenated SPN (SPN-oxy) and SPN-oxy-Hb in deoxygenated PBS for 60 min. (**f**) Detection of ·OH generation in SPN-Hb and SPN-Hb@RBCM using Fenton-like reaction. (**g**) In vivo NIR-II fluorescence images of tumor-bearing mice after intravenous injection of SPN-Hb and SPN-Hb@RBCM. (**h**) Fluorescence images of excised major organs and tumors from tumor-bearing mice in panel. (**d**–**h**) Reprinted with permission from ref. [78]. Copyright American Chemical Society publishers.

The integration of blood cell membrane-coated nanomaterials into PDT represents a cutting-edge advancement in tumor treatment. Specific physiological attributes of the RBC membrane can be explored for encapsulating PS materials, aiming to attain enhanced therapeutic effects. Li and co-workers found that magnetic mesoporous silica nanoparticles coated with RBC membrane were able to achieve high accumulation in tumors induced by the magnetic field (Figure 3a) [74]. Mesoporous silica nanoparticles with a diameter of 80 nm were prepared by embedding magnetic colloids into the micropore structure; then the PS (Hypocrellin B, HB) was also embedded into the micropore structure, and then mixed with freshly extracted RBC membrane to obtain RBC@MMSN-HB. After being wrapped in RBC membranes, the absorption peak of HB does not shift after ultraviolet irradiation, and the HB content in the body will not be lost on a large scale. After irradiation, singlet oxygen (^1^O_2_) was produced. Additional experiments in mice demonstrated that the circulation time of mesoporous silica particles was significantly extended when coated with an RBC membrane, irrespective of HB loading. This result underscores the effectiveness of RBC membrane coating as a biological camouflage strategy. At the same time, the weight of the tumor in mice was reduced the most under the synergistic treatment of RBC@MMSN-HB, PDT, and magnetic field, showing a good tumor treatment effect. It is worth mentioning that other blood cells may have some roles in the tumor treatment process, so we can use the blood cell membrane to extend the blood circulation time, and also combine it with other blood cells at the same time in order to play a better role in tumor treatment [75] (Figure 3b). In another study, Pei et al. reported the idea of combining chemotherapy and PDT in the treatment of tumors by wrapping a nanoparticle kernel composed of paclitaxel dimer (PTX_2_-TK) and 5, 10, 15, 20-tetraphenyl chlorin (TPC) with RBC membrane (Figure 3c). Under proper light irradiation, TPC can produce enough ROS to kill tumor cells. Meanwhile, it can also trigger the disintegration of PTX_2_-TK and release paclitaxel, further enhancing the therapeutic effect of tumors [76]. Recently, Zhang and co-workers studied the anoxic environment inside the tumor and explored corresponding improvement plans.

RBCs rely on hemoglobin (Hb) to transport oxygen, and the red blood cell-based materials (mmRBCs) can load more Hb than natural RBCs, allowing them to carry more oxygen. The addition of polydopamine (PDA) to mmRBCs acts as an antioxidant enzyme that can prevent the oxidation of Hb during its circulation in the body. At the same time, PDA can increase the drug loading and PS loading, and improve the anoxic environment of the tumor site during the PDT treatment [77]. Zeng et al. utilized semiconductor polymer nanoparticles (SPNs) covalently conjugated with hemoglobin (Hb) and camouflaged them using red blood cell membranes to obtain the SPN-Hb@RBCM. This strategy extended the systemic circulation time of the nanoparticles and facilitated their accumulation within tumors [78]. In the in vitro assessment of ROS generation and oxygen release capacity, both SPN and SPN-Hb showed some single-linear oxygen generation capacity. Specifically, the single-linear state oxygen production capacity of the SPN-Hb group was significantly better than that of the SPN group. This property did not produce a detectable change after wrapping the RBC membrane. (Figure 3d,e). Comparing the oxygen release behavior of SPN and SPN-oxy-Hb, it was determined that SPN-Hb@RBCM NPs carry approximately 0.142 μg of oxygen per 1 mg (Figure 3f). In vivo fluorescence imaging in mice revealed that the fluorescence signal of SPN appeared at tumor sites around 2 h post-administration, showing time-dependent accumulation. The signal intensity peaked at 12 h, indicating this as the optimal time for PDT treatment (Figure 3g,h). Compared to other experimental groups, the SPN-Hb@RBCM group exhibited the most pronounced tumor growth inhibition and ablation effects under laser irradiation.

**Figure 4:**
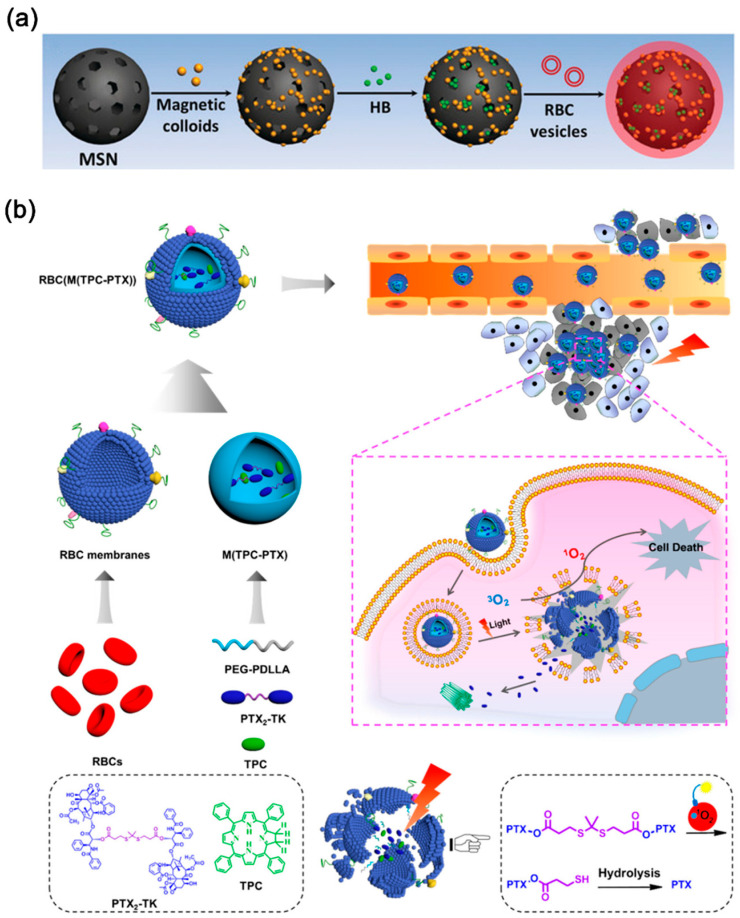
(**a**) Schematic illustration of AmmRBCs for tumor therapy. (I) A mmRBCs accumulate in the tumor site and boost ^1^O_2_ generation for enhanced PDT. (II) PDA in AmmRBC functions like catalase and superoxide dismutase in RBC to protect Hb from oxidant damage during circulation. Reprinted with permission from ref. [74]. Copyright Wiley Publishers. (**b**) Schematic illustration of RBC membrane-camouflaged dimeric prodrug NPs (RBC(M(TPC-PTX))). Reprinted with permission from ref. [76]. Copyright American Chemical Society publishers.

The application of platelet- and white blood cell-coated nanomaterials in antitumor therapy is also attracting attention. For example, Yang et al. prepared red cell membrane and platelet membrane-coated poly lactic-co-glycolic acid NPs loaded with Verteporfin [79]. The cell internalization was compared with that of tumor cells and fibroblasts, and it was found that platelet NPs were more efficient in being internalized by tumor cells than RBC membrane-coated NPs, and could actively target fibroblasts (Figure 4a). Verteporfin NPs coated with platelet membrane exhibited reduced accumulation in the liver and lung compared to those coated with RBC membrane. Records of in vivo tumor treatment demonstrated that platelet membrane-coated nanoparticles exhibited superior therapeutic efficacy in mice compared to RBC membrane coatings, with the inhibition persisting for an extended duration. In another study, IR780 as a PS was encapsulated in a platelet membrane with metformin to form nanoparticles and delivered to the tumor microenvironment, which can effectively inhibit immunogenic activation and produce a large number of T lymphocytes, control tumor metastasis, and ablate the primary tumor. It was found that metformin inhibits mitochondrial respiration in cancer cells, ameliorating the tumor’s hypoxic environment, thereby increasing oxygen availability for PDT and consequently enhancing its efficacy [80].

Presently, the majority of antitumor drugs utilizing platelets or materials coated with platelet membranes as drug delivery systems leverage the attributes of cytokines, glycoproteins, and antibodies present on the platelet surface for anti-tumor and targeted therapy. However, limited research has focused on modifying or optimizing the membrane surface (Figure 4b). Reported platelet medications currently pose bleeding risks due to their non-selective inhibition of thrombosis. Using nanotechnology to deliver platelet-suppressing drugs directly into tumors can mitigate side effects on the clotting system [75]. The role of platelets in cancer development and their impact on tumor cells’ mechanisms remain controversial in clinical studies. Additionally, there is a scarcity of extensive clinical studies to comprehend and validate the interactions between cancer cells and platelets in various cancer types. Addressing these existing issues requires further research to optimize the utilization of platelet-related materials in antitumor therapy [81].

Inflammation occurs when the body responds immunologically to risk factors such as pathogens, injuries, and irritants. The immune system rapidly activates to eliminate pathogens and facilitate tissue repair, with white blood cells playing a pivotal role. Neutrophils comprise 50–70% of the circulating white blood cells, representing the highest proportion, and they predominantly accumulate at inflammation sites. However, stimulating the accumulation of white blood cells in the same site repeatedly, over a long period, induces the sustained release of pro-inflammatory molecules, triggering inflammatory disorders and many inflammatory diseases [82,83,84]. There are two subgroups of neutrophils in the tumor inflammatory microenvironment: one is type 1 neutrophils (N1), which have antibacterial and anti-tumor properties, and the other is type 2 neutrophils, which can promote tumor function [85]. Meanwhile, the inflammatory response worsens the hypoxia of the tumor microenvironment and decreases pH, which is not conducive to the treatment and drug efficacy of PDT [86]. Based on these studies, one can use the rapid inflammation response and immune escape properties of white blood cells to combine with nanotherapeutic drugs and therapies to treat inflammation and cancer. Similarly to the nanotechnology used by RBCs and platelets, leukocytes can be coupled with polylactic glycolic acid (PLGA), PEG, liposomes, etc., to improve leukocyte targeting and drug transfer rate, and prolong cycle time [87,88]. For example, Kang et al. demonstrated that nanoparticles coated with neutrophil membrane can effectively consume circulating tumor cells, and have certain inhibitory effects on early metastasis [89]. The surface of PLGA nanoparticles was coated with a neutral cell membrane so that the proteins on the surface of the cell membrane were not destroyed. In comparison to uncoated nanoparticles, it exhibits a higher affinity for binding to cancer cells in vitro and an improved capacity to capture circulating tumor cells in vivo. When loaded with carfilzomib, a proteasome inhibitor, circulating tumor cells in the blood can be selectively consumed. Mouse experiments demonstrated that drug-loaded nanoparticles coated with neutrophils effectively inhibited the formation of tumors from metastasizing 4T1 cells in lymph nodes, while also displaying an inhibitory effect on lung metastasis in tumors.

In conclusion, blood cell membrane-coated nanomaterials represent a promising frontier in the realm of PDT for tumor treatment. Their unique ability to leverage the natural properties of blood cells enhances biocompatibility and facilitates targeted delivery, thereby improving therapeutic efficacy while minimizing off-target effects. Despite the progress made in understanding their potential, significant gaps remain in the exploration of their mechanisms of action, particularly concerning the influence of external factors such as pH and redox conditions. Future research should focus on elucidating these interactions and optimizing the design of these nanomaterials to fully exploit their capabilities in PDT. By systematically addressing these challenges, we can pave the way for the development of innovative therapeutic strategies that leverage the unique properties of blood cell membranes in the battle against tumors.

## 5. Application of Blood Cell Membrane-Coated Nanomaterials in Combined Therapy of Tumors

**Figure 5:**
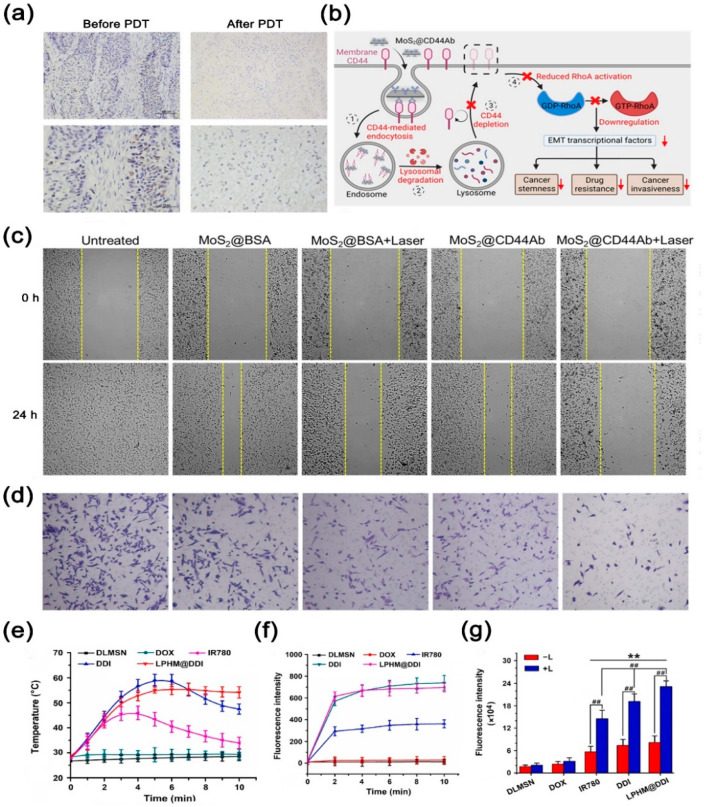
(**a**) Changes in the pathological tissue TUNEL staining 3 months later after PDT. Reprinted with permission from ref. [66]. Copyright Springer Publishers. (**b**) Schematic illustrating the mechanism of how MoS_2_@CD44Ab + laser treatment affects the CD44-regulated EMT signaling pathway, thus causing the attenuation of stemness, drug resistance, and invasiveness. (**c**,**d**) Anti-invasive and migratory effects of MoS_2_@CD44Ab and MoS_2_@CD44Ab + Laser groups on cancer cells. (**b**–**d**) Reprinted with permission from ref. [94]. Copyright American Chemical Society publishers. (**e**) In vitro photothermal performance of LPHM@DDI NPs (2 W/cm^2^, 808 nm). (**f**,**g**) Levels of ROS production at the cellular level by IR780, DDI NPs, and LPHM@DDI NPs. ** represents *p* < 0.01 vs. control; ## represents *p* < 0.01 between two groups. (**e**–**g**) Reprinted with permission from ref. [95]. Copyright Elsevier Publishers.

Tumor treatment involves surgical resection, radiotherapy, chemotherapy, targeted therapy, PDT, PTT, and other therapeutic methods, which have been widely explored. However, a singular approach is insufficient to address diverse tumor types. Due to the intricate microenvironment of tumor tissue, rapid metastasis, and varying degrees of drug-induced damage to the human body, a comprehensive solution is yet to be found. Therefore, in clinical treatment, combination therapy should be carried out. Liu and colleagues separately loaded DOX and Ce6 with reduced graphene oxide (rGO) to test their therapeutic effects in the same 2D and 3D model conditions. The results showed that nanocarrier-based PTT has good penetration dependence, which is superior to chemotherapy and PDT under the same experimental conditions, and has great advantages for the treatment of local tumors such as breast tumors and melanoma [90]. The individual therapeutic efficacy of chemotherapy and PDT is less potent than that of PTT due to the influence of fibroblasts and the extracellular matrix. Some clinical case studies have indicated that PDT alone yields better short-term therapeutic outcomes in primary bronchopulmonary cancer treatment [66] (Figure 5a). However, combining PDT with chemotherapy and molecularly targeted drugs enhances its efficacy, reduces drug resistance, and minimizes adverse effects on the body [91]. These examples also serve to highlight the importance of combination therapy as a key research direction in antitumor therapy [92].

### 5.1. Combined PTT and PDT

Yang and colleagues found that the combination of PTT and PDT can be achieved by irradiating the same material with different NIR wavelengths [93]. Chlorin e6 (Ce6) was first embedded in the blood cell membrane, which didn’t break the integrity of the blood cell membrane. Then, the Prussian blue nanoparticles were wrapped by the blood cell membrane containing Ce6. Under the irradiation of a 660 nm laser, Ce6 can mediate the therapeutic effect of PDT. At this time, the vesicles of the RBC membrane will disintegrate rapidly. When the laser wavelength was 808 nm, Prussian blue nanoparticles exerted the PTT effect to achieve the effect of PTT and PDT combined therapy. In addition, controlling the heating interval at the infrared irradiation site can disrupt undifferentiated tumor cells [94] (Figure 5b). It was found that infrared irradiation can be used to drive phenotypic changes and differentiation of tumor stem cell-like cells, reducing stemness, migration, and drug resistance of cancer cells (Figure 5c,d). Wang and co-workers developed a novel phototherapy/chemotherapy agent for the treatment of triple-negative breast cancer, featuring efficient loading and targeted delivery capabilities [95]. The study initially involved the fabrication of dendritic large-pore mesoporous silica nanoparticles (DLMSNs), which were subsequently co-loaded with near-infrared fluorescence dye IR780 and chemotherapeutic agent DOX into the large pores (DLMSN@DOX/IR780, DDI). These NPs were then enveloped with a hybrid membrane of white blood cell and platelet LPHM to yield LPHM@DDI NPs. Facilitated by LPHM-mediated targeting, LPHM@DDI NPs exhibited exceptional TNBC-targeting capability and demonstrated outstanding PDT/PTT performance. Following NIR laser irradiation, LPHM@DDI NPs demonstrated synergistic cytotoxicity and apoptosis induction in triple-negative breast cancer cells, inhibiting angiogenesis and inducing tumor ablation to suppress mouse tumor growth and recurrence. In vitro photothermal experiments revealed that LPHM@DDI NPs could achieve temperatures exceeding 55 °C within 5 min and maintain relatively stable temperatures over time (Figure 5e). Flow cytometry analysis indicated significantly higher levels of ROS in cells treated with LPHM@DDI NPs compared to other experimental groups (Figure 5f,g).

### 5.2. Synergistic Effects of Immunotherapy and PDT

Immunotherapy involves stimulating the patient’s immune system to suppress the primary tumor, tumor metastasis, and recurrence [96,97]. Immunotherapy has been employed using RBC membrane-encapsulated PLGA NPs, wherein the RBC membrane offers effective protection against antigenic peptides and Toll-like receptor 4 agonists [27]. The proposed mechanism of action for NP-based immunotherapy involves the activation of antigen-specific CD8 T cells, leading to the generation of cytotoxic T lymphocytes that target and eliminate cancer cells. This approach synergizes the antigen sequestration and stimulatory properties of polymeric NPs with the antigen presentation and adjuvant functions of RBCs, potentially yielding improved therapeutic outcomes in melanoma. Li et al. combined the triple-negative breast cancer treatment drug paclitaxel (PTX) with indocyanine green to produce nanoparticles [98]. PTX can modulate the immunosuppressive tumor microenvironment, augment the immune checkpoint blockade, exert an immune-regulating function, and impede the mitosis of cancer cells, thereby inhibiting tumor growth. The self-assembly of PTX NPs and indocyanine green can bind to the immune checkpoint inhibitor αPD-L_1_ to induce a memory immune response and inhibit tumor recurrence and metastasis in triple-negative breast cancer mice. Combined with PDT, the infiltration area of cytotoxic T lymphocytes in the tumor was increased, and the tumor metastasis was effectively inhibited, showing a satisfactory antitumor effect. Inspired by the idea of immunotherapy and endogenous cell membrane encapsulation of nanoparticles, MnO_x_/PDA nanobombs were prepared using the nano-casting method, and macrophage membranes were encapsulated in the outer layer of the nanobombs [99]. Macrophages provide tumor targeting and exert photothermal and photodynamic properties for synergistic therapy after entering the tumor MnO_x_/PDA. The expression of markers and regulatory factors related to tumor stem cell-like cells showed an attenuated trend after treatment, and the inhibition rate of the tumor reached 70.8% in a colorectal cancer model.

### 5.3. Combined Phototherapy and Chemotherapy

Chemotherapeutic agents are typically cytotoxic, disrupting the growth and proliferation of cancer cells by damaging DNA, inhibiting cell division, influencing specific phases of the cell cycle, and targeting particular molecules or pathways in cancer cells through targeted therapy [100,101]. However, these drugs also affect normal cells, leading to more severe side effects in patients [102]. Consequently, the research, development, and application of nanomaterials derived from blood cells offer significant potential to enhance the biocompatibility of chemotherapeutic agents. The unique structural properties of these nanomaterials can improve drug delivery efficiency by effectively encapsulating and protecting the therapeutic agents, thereby enhancing their solubility and stability. This optimization ensures that drugs reach the effective concentration at the tumor site. Furthermore, the surface of biomimetic nanomaterials, such as blood cell-coated nanoparticles, can be modified with various substances to enable personalized treatment and promote an immune response [103]. Wu et al. developed a vascular-specific drug delivery strategy targeting the tumor vasculature, a critical area for cancer therapy [104]. They engineered thrombin/tirapazamine@HRBCs, which release their payloads precisely within the tumor vasculature upon laser irradiation. The thrombin encapsulated within the red blood cells induces specific occlusion of the tumor vasculature, resulting in tumor deoxygenation, which further activates the synergistic cytotoxicity of tirapazamine against surrounding tumor cells. This strategy capitalizes on the excellent biocompatibility and extended circulation time of red blood cells, enabling precise drug release at the tumor site and addressing the challenge of targeted chemotherapy. However, this approach’s reliance on laser-induced drug release poses limitations, particularly in achieving effective anti-tumor effects for tumors located in deeper organs. Similarly, Xie and colleagues developed a MoS_2_-RBC drug delivery system (MoS_2_-RBC-DOX), facilitating both targeted drug delivery and PTT. The MoS_2_-RBC-DOX system demonstrated pH-dependent release behavior in the tumor microenvironment, with a maximum DOX release of 45.80% at pH 5.0, which is double that observed in a weakly alkaline environment. This system exploits the targeting effect of chemotherapeutic agents on tumors. Moreover, after near-infrared light irradiation, the release rate of DOX was significantly accelerated, and the peak release time was reduced [105]. Modification of the RBC membrane enhanced the stability of MoS_2_ and decreased its toxicity, allowing for more precise and rapid drug release at the tumor site, resulting in a stronger cytotoxic effect on cancer cells. While these anti-tumor strategies have inherent limitations, they represent promising advancements in the application of chemotherapy.

The following table lists the effectiveness and promising applications of these nanomaterials in different tumor types (Table 1).

## 6. Conclusions and Outlooks

The rapid development of nanotechnology provides new opportunities for anti-tumor strategies. As a multifunctional biomimetic nanoplatform, blood cell membrane-coated nanomaterials are increasingly becoming a research hotspot due to their excellent biocompatibility, targeting, and sustained release properties. By cleverly exploiting the natural structure of blood cell membranes, these nanomaterials can not only effectively prolong the circulation time in vivo, but also significantly enhance the efficacy of PTT and PDT. The application of blood cell membranes gives nanomaterials the advantage of avoiding clearance by the immune system, thus enhancing their retention and drug delivery efficiency in the tumor microenvironment. In particular, blood cell membranes are enriched with specific antibodies and antigens that enable precise targeting of tumor cells, greatly improving therapeutic selectivity. In addition, the fusion of nanomaterials with blood cell membranes endows them with unique physicochemical properties, enabling optimized performance in terms of photothermal conversion and photosensitivity. Currently, researchers have developed several different types of blood cell membrane-coated nanomaterials, including combinations based on carbon, metals, and other biomaterials. These novel nanomaterials not only exhibit good drug-loading capacity but also enable smart responses to the tumor microenvironment by modulating their physicochemical properties. Combining these advantages, future research could focus on how to further improve the targeting and efficacy of nanomaterials and explore their potential application in combination therapies. Although there are many research reports on the application of blood cell membrane-encapsulated nanomaterials for tumor therapy, most of them remain in the laboratory research stage, and none of the materials has even been applied in clinical phase III studies. Therefore, in addition to continuing to expand the types of blood cell membrane-coated materials, more research is needed to address the problems faced during clinical translation.

Integrating current treatment methods with state-of-the-art tumor treatment mechanisms, ongoing explorations, and research experiments on tumors offer new insights to effectively address complex cancers. In addition to blood cell membrane-encapsulated NPs, other types of cell membranes, such as those from neutrophils, macrophages, and cancer cells, have also been explored in antitumor therapy [101,102,103]. Neutrophil membranes exhibit inherent chemotactic and immunomodulatory functions, enabling effective migration to the tumor microenvironment [119,120]. In this setting, they play a crucial role in regulating the tumor milieu and enhancing the antitumor immune response through the release of cytokines and chemokines. However, the short lifespan of neutrophils, coupled with the heterogeneity and complexity of their isolation processes, limits their utility as consistent modifiers of NPs. Additionally, the function and phenotype of neutrophils can vary significantly among individuals, which may influence their reliability and effectiveness in tumor treatment. On the other hand, tumor-associated macrophages, known for their accumulation in tumor tissues, present an opportunity for tumor-targeted therapies when employing macrophage membrane-encapsulated NPs [121]. Furthermore, cancer cell membranes possess distinct immune evasion properties, which allow encapsulated NPs to evade detection and rejection by the immune system, thus enhancing therapeutic efficacy [122]. When considering the advantageous properties of these membranes, blood cell membrane-encapsulated NPs emerge as particularly promising. They offer excellent biocompatibility, prolonged circulation times, versatility in modification, and enhanced targeting capabilities, all while providing a more cost-effective preparation method. These attributes position blood cell membrane-encapsulated NPs as a significant focus for further research into biomimetic nanomaterials for cancer therapy.

With the development of gene editing and synthetic biology, the transformation of blood cell membranes into targeting carriers with specific functions will provide a new direction for personalized tumor therapy. Meanwhile, based on multimodal imaging technology, combining PTT and PDT with other therapeutic means, such as chemotherapy or immunotherapy, is expected to achieve better therapeutic effects. Therefore, blood cell membrane-coated nanomaterials, as a multifunctional biomimetic nano platform, not only provide new ideas for anti-tumor therapy but also lay the foundation for achieving the goal of precision medicine. Through continuous research and innovation, these nanomaterials will play an indispensable role in future tumor therapy, helping to improve the survival rate and quality of life of patients.

## Figures and Tables

**Table 1:** Examples of membrane-encapsulated nanomaterials for tumor therapy.

	Membrane Coating	Materials	Applications	Advantage *	Number
**PTT**	RBC membranes and platelet membranes	PPy@[R-P] NPs	Micro thrombosis sites	I, II	[56]
	RBC membranes	RBC-AuNCs	Prolonging the circulation lifetime	I	[52]
		IR780@rRBC NPs	Drug delivery platform	I	[53]
		RBC-MNs	Imaging-guided cancer therapy	I	[57]
		Melanin@RBC	Enhanced PTT	I	[58]
		PB@RBC/Ce6 NPs	Effect in boosting the necrosis of tumor cells	I	[93]
		Cyp-MNC@RBCs	Increased stability and dispersion	I	[106]
		MoS2-RBC-DOX	Breast cancer	IV	[107]
		RHAuNCs-miRNA	Improved miRNA delivery	I	[108]
		M/B@R	Nasopharyngeal carcinoma	I, IV	[109]
	Cancer cell membrane	4T1-HANG-GNR-DC	Tumor targeting therapy	I, II	[110]
**PDT**	Cancer cell membrane	CM/SLN/Ce6	Gastric cancer	I, II	[111]
		CM-MMNPs	Tumor targeting therapy and magnetic resonance imaging	I, II	[112]
		CDAuNs	Breast cancer	II	[113]
		CCNP	Drug delivery	II	[114]
	RBC membranes	RBC@MMSNs	Cancer treatment	I–III	[74]
		RBC(M(TPC-PTX))	Cancer treatment	I	[76]
		AmmRBC	Cancer treatment	I, III	[77]
		UCNPs@mSiO2	Inhibition of tumor growth	I, III, IV	[115]
	Platelet membranes	PM-IR780-Met NPs	Cancer treatment	I, III	[80]
	Neutrophil membranes	NM-NP-CFZ	Treat cancer metastasis	III	[89]
**Combination therapy**	RBC membranes	PB@RBC/Ce6	Cancer treatment	I	[93]
	Leukocyte/platelet hybrid membranes	LPHM@DDI	Cancer treatment	I–III	[95]
	Macrophage membrane	MnOx/PDA	Colorectal cancer	I	[99]
**Other treatments**	Platelet membrane	PltM@Crb-P beta AE-ALG NPs	Chemotherapy	II	[116]
		AuNCs-PEG-SNP-PM	NO therapy/radiotherapy	I, II	[117]
	White blood cell membrane	GCMNPs	Ferrotherapy/immunotherapy	II, III	[118]

* I: long circulation times; II: targeting properties, III: immune escapable, IV: increased NPs accumulation.

## References

[1] Hao X.Y., Wu J.Y., Xiang D.X., Yang Y.Y. (2022). Recent advance of nanomaterial-mediated tumor therapies in the past five years. Front. Pharmacol..

[2] Alfei S., Schito G.C., Schito A.M., Zuccari G. (2024). Reactive Oxygen Species (ROS)-Mediated Antibacterial Oxidative Therapies: Available Methods to Generate ROS and a Novel Option Proposal. Int. J. Mol. Sci..

[3] Malik D., Mahendiratta S., Kaur H., Medhi B. (2021). Futuristic approach to cancer treatment. Gene.

[4] Gai S.L., Yang G.X., Yang P.P., He F., Lin J., Jin D.Y., Xing B.G. (2018). Recent advances in functional nanomaterials for light-triggered cancer therapy. Nano Today.

[5] Liu C., Zhang S.B., Li J.H., Wei J., Mullen K., Yin M.Z. (2019). A water-soluble, NIR-absorbing quaterrylenediimide chromophore for photoacoustic imaging and efficient photothermal cancer therapy. Angew. Chem. Int. Ed..

[6] Liu L., Zhang S., Zhao L., Gu Z., Duan G., Zhou B., Yang Z., Zhou R. (2018). Superior compatibility of C_2_N with human red blood cell membranes and the underlying mechanism. Small.

[7] Harris J.M., Chess R.B. (2003). Effect of pegylation on pharmaceuticals. Nat. Rev. Drug Discov..

[8] Shi L.W., Zhang J.Q., Zhao M., Tang S.K., Cheng X., Zhang W.Y., Li W.H., Liu X.Y., Peng H.S., Wang Q. (2021). Effects of polyethylene glycol on the surface of nanoparticles for targeted drug delivery. Nanoscale.

[9] Yang S., Han G.H., Chen Q., Yu L., Wang P., Zhang Q., Dong J., Zhang W., Huang J.X. (2021). Au-Pt nanoparticle formulation as a radiosensitizer for radiotherapy with dual effects. Int. J. Nanomed..

[10] Farshbaf M., Valizadeh H., Panahi Y., Fatahi Y., Chen M.W., Zarebkohan A., Gao H.L. (2022). The impact of protein corona on the biological behavior of targeting nanomedicines. Int. J. Pharm..

[11] Xu W.W., Xu M.Y., Xiao Y.M., Yu L., Xie H.R., Jiang X.H., Chen M.W., Gao H.L., Wang L. (2022). Changes in target ability of nanoparticles due to protein corona composition and disease state. Asian J. Pharm. Sci..

[12] Xiao W., Wang Y.Z., Zhang H.L., Liu Y.W., Xie R., He X.Q., Zhou Y., Liang L.Q., Gao H.L. (2021). The protein corona hampers the transcytosis of transferrin-modified nanoparticles through blood-brain barrier and attenuates their targeting ability to brain tumor. Biomaterials.

[13] Oroojalian F., Beygi M., Baradaran B., Mokhtarzadeh A., Shahbazi M.A. (2021). Immune Cell Membrane-Coated Biomimetic Nanoparticles for Targeted Cancer Therapy. Small.

[14] Luo G.F., Chen W.H., Zeng X., Zhang X.Z. (2021). Cell primitive-based biomimetic functional materials for enhanced cancer therapy. Chem. Soc. Rev..

[15] Liu W.C., Yan Q.W., Xia C., Wang X.X., Kumar A., Wang Y., Liu Y.W., Pan Y., Liu J.Q. (2021). Recent advances in cell membrane coated metal-organic frameworks (MOFs) for tumor therapy. J. Mater. Chem. B.

[16] Yang L.B., Liu Q., Zhang X.Q., Liu X.W., Zhou B.X., Chen J.N., Huang D., Li J.Q., Li H.L., Chen F. (2020). DNA of neutrophil extracellular traps promotes cancer metastasis via CCDC25. Nature.

[17] Jiang Q., Wang K., Zhang X.Y., Ouyang B.S., Liu H.X., Pang Z.Q., Yang W.L. (2020). Platelet Membrane-Camouflaged Magnetic Nanoparticles for Ferroptosis-Enhanced Cancer Immunotherapy. Small.

[18] Xie J.B., Shen Z.Y., Anraku Y., Kataoka K., Chen X.Y. (2019). Nanomaterial-based blood-brain-barrier (BBB) crossing strategies. Biomaterials.

[19] Qin M., Du G.S., Sun X. (2020). Biomimetic cell-derived nanocarriers for modulating immune responses. Biomater. Sci..

[20] Zhu C., Ma J., Ji Z., Shen J., Wang Q. (2021). Recent advances of cell membrane coated nanoparticles in treating cardiovascular disorders. Molecules.

[21] Yoshida T., Prudent M., D’Alessandro A. (2019). Red blood cell storage lesion: Causes and potential clinical consequences. Blood Transfus..

[22] Liang X., Ye X.Y., Wang C., Xing C.Y., Miao Q.W., Xie Z.J., Chen X.L., Zhang X.D., Zhang H., Mei L. (2019). Photothermal cancer immunotherapy by erythrocyte membrane-coated black phosphorus formulation. J. Control. Release.

[23] Xia Q., Zhang Y.T., Li Z., Hou X.F., Feng N.P. (2019). Red blood cell membrane-camouflaged nanoparticles: A novel drug delivery system for antitumor application. Acta Pharm. Sin. B.

[24] Sevencan C., McCoy R.S.A., Ravisankar P., Liu M., Govindarajan S., Zhu J.Y., Bay B.H., Leong D.T. (2020). Cell Membrane Nanotherapeutics: From Synthesis to Applications Emerging Tools for Personalized Cancer Therapy. Adv. Ther..

[25] Qin X., Zhu L., An X., Zhang C., Li J.W., Yan F., Zhang W.J., Qu K., Zhang K., Wu W. (2024). Hybrid cell membranes camouflaged copper-loaded nano-prodrug for tumor angiogenesis inhibition and cell cuproptosis. Chem. Eng. J..

[26] Li M., Fang H., Liu Q., Gai Y., Yuan L., Wang S., Li H., Hou Y., Gao M., Lan X. (2020). Red blood cell membrane-coated upconversion nanoparticles for pretargeted multimodality imaging of triple-negative breast cancer. Biomater. Sci..

[27] Guo Y.Y., Wang D., Song Q.L., Wu T.T., Zhuang X.T., Bao Y.L., Kong M., Qj Y., Tan S.W., Zhang Z.P. (2015). Erythrocyte Membrane-Enveloped Polymeric Nanoparticles as Nanovaccine for Induction of Antitumor Immunity against Melanoma. Acs Nano.

[28] Yang J., Miao X.Y., Guan Y., Chen C.J., Chen S.F., Zhang X.M., Xiao X., Zhang Z., Xia Z.Y., Yin T.H. (2021). Microbubble Functionalization with Platelet Membrane Enables Targeting and Early Detection of Sepsis-Induced Acute Kidney Injury. Adv. Healthc. Mater..

[29] Fan M.L., Jiang M.X. (2020). Core-shell nanotherapeutics with leukocyte membrane camouflage for biomedical applications. J. Drug Target..

[30] Kunde S.S., Wairkar S. (2021). Platelet membrane camouflaged nanoparticles: Biomimetic architecture for targeted therapy. Int. J. Pharm..

[31] Wang S.Y., Duan Y.O., Zhang Q.Z., Komarla A., Gong H., Gao W.W., Zhang L.F. (2020). Drug Targeting via Platelet Membrane-Coated Nanoparticles. Small Struct..

[32] Rao L., Bu L.L., Meng Q.F., Cai B., Deng W.W., Li A., Li K.Y., Guo S.S., Zhang W.F., Liu W. (2017). Antitumor Platelet-Mimicking Magnetic Nanoparticles. Adv. Funct. Mater..

[33] Török O., Schreiner B., Schaffenrath J., Tsai H.C., Maheshwari U., Stifter S.A., Welsh C., Amorim A., Sridhar S., Utz S.G. (2021). Pericytes regulate vascular immune homeostasis in the CNS. Proc. Natl. Acad. Sci. USA.

[34] Friebel E., Kapolou K., Unger S., Núñez N.G., Utz S., Rushing E.J., Regli L., Weller M., Greter M., Tugues S. (2020). Single-Cell Mapping of Human Brain Cancer Reveals Tumor-Specific Instruction of Tissue-Invading Leukocytes. Cell.

[35] Yang L., Yuan M., Ma P.A., Chen X.R., Cheng Z.Y., Lin J. (2023). Assembling AgAuSe Quantum Dots with Peptidoglycan and Neutrophils to Realize Enhanced Tumor Targeting, NIR (II) Imaging, and Sonodynamic Therapy. Small Methods.

[36] Liu Y., Luo J.S., Chen X.J., Liu W., Chen T.K. (2019). Cell Membrane Coating Technology: A Promising Strategy for Biomedical Applications. Nano Micro Lett..

[37] Zou S.J., Wang B.L., Wang C., Wang Q.Q., Zhang L.M. (2020). Cell membrane-coated nanoparticles: Research advances. Nanomedicine.

[38] Zhang Q.Z., Zhou J.L., Zhou J.R., Fang R.H., Gao W.W., Zhang L.F. (2021). Lure-and-kill macrophage nanoparticles alleviate the severity of experimental acute pancreatitis. Nat. Commun..

[39] Mohale S., Kunde S.S., Wairkar S. (2022). Biomimetic fabrication of nanotherapeutics by leukocyte membrane cloaking for targeted therapy. Colloids Surf. B Biointerfaces.

[40] Li X.S., Lovell J.F., Yoon J., Chen X.Y. (2020). Clinical development and potential of photothermal and photodynamic therapies for cancer. Nat. Rev. Clin. Oncol..

[41] Sun W., Zhong G., Kubel C., Jelle A.A., Qian C.X., Wang L., Ebrahimi M., Reyes L.M., Helmy A.S., Ozin G.A. (2017). Size-tunable photothermal germanium nanocrystals. Angew. Chem. Int. Ed..

[42] Zhang X.F., Liu Z.G., Shen W., Gurunathan S. (2016). Silver nanoparticles: Synthesis, characterization, properties, applications, and therapeutic approaches. Int. J. Mol. Sci..

[43] Yang K., Zhang S.A., Zhang G.X., Sun X.M., Lee S.T., Liu Z.A. (2010). Graphene in mice: Ultrahigh in vivo tumor uptake and efficient photothermal therapy. Nano Lett..

[44] Huang X.H., El-Sayed I.H., Qian W., El-Sayed M.A. (2006). Cancer cell imaging and photothermal therapy in the near-infrared region by using gold nanorods. J. Am. Chem. Soc..

[45] Vijayan V., Uthaman S., Park I.K., Noh I. (2018). Cell Membrane Coated Nanoparticles: An Emerging Biomimetic Nanoplatform for Targeted Bioimaging and Therapy. Biomimetic Medical Materials: From Nanotechnology to 3d Bioprinting.

[46] Mathiyazhakan M., Wiraja C., Xu C.J. (2018). A concise review of gold nanoparticles-based photo-responsive liposomes for controlled drug delivery. Nano Micro Lett..

[47] Gao C.Y., Lin Z.H., Jurado-Sánchez B., Lin X.K., Wu Z.G., He Q. (2016). Stem cell membrane-coated nanogels for highly efficient In vivo tumor targeted drug dselivery. Small.

[48] Vaupel P., Harrison L. (2004). Tumor hypoxia: Causative factors, compensatory mechanisms, and cellular response. Oncologist.

[49] Kumar S.T., Donzelli S., Chiki A., Syed M.M.K., Lashuel H.A. (2020). A simple, versatile and robust centrifugation-based filtration protocol for the isolation and quantification of α-synuclein monomers, oligomers and fibrils: Towards improving experimental reproducibility in α-synuclein research. J. Neurochem..

[50] Lobb R.J., Becker M., Wen S.W., Wong C.S.F., Wiegmans A.P., Leimgruber A., Moller A. (2015). Optimized exosome isolation protocol for cell culture supernatant and human plasma. J. Extracell. Vesicles.

[51] Zhao J.N., Ruan J., Lv G.Y., Shan Q., Fan Z.P., Wang H.B., Du Y., Ling L.B. (2022). Cell membrane-based biomimetic nanosystems for advanced drug delivery in cancer therapy: A comprehensive review. Colloids Surf. B Biointerfaces.

[52] Piao J.G., Wang L.M., Gao F., You Y.Z., Xiong Y.J., Yang L.H. (2014). Erythrocyte membrane is an alternative coating to polyethylene glycol for prolonging the circulation lifetime of gold nanocages for photothermal therapy. Acs Nano.

[53] Wu P.K., Jiang X., Yin S., Yang Y., Liu T.Q., Wang K.K. (2021). Biomimetic recombinant of red blood cell membranes for improved photothermal therapy. J. Nanobiotechnology.

[54] Ye J.T., Yu Y.L., Li Y.J., Yao B., Gu M.E., Li Y., Yin S.C. (2024). Nanoparticles Encapsulated in Red Blood Cell Membranes for Near-Infrared Second Window Imaging-Guided Photothermal-Enhanced Immunotherapy on Tumors. Acs Appl. Mater. Interfaces.

[55] Pei W.J., Huang B.Y., Chen S.J., Wang L., Xu Y., Niu C.C. (2020). Platelet-Mimicking Drug Delivery Nanoparticles for Enhanced Chemo-Photothermal Therapy of Breast Cancer. Int. J. Nanomed..

[56] Liu Y., Wang X.J., Ouyang B.S., Liu X.P., Du Y., Cai X.Z., Guo H.S., Pang Z.Q., Yang W.L., Shen S. (2018). Erythrocyte-platelet hybrid membranes coating polypyrrol nanoparticles for enhanced delivery and photothermal therapy. J. Mater. Chem. B.

[57] Rao L., Cai B., Bu L.L., Liao Q.Q., Guo S.S., Zhao X.Z., Dong W.F., Liu W. (2017). Microfluidic electroporation-facilitated synthesis of erythrocyte membrane-coated magnetic nanoparticles for enhanced imaging-guided cancer therapy. Acs Nano.

[58] Jiang Q., Luo Z.M., Men Y.Z., Yang P., Peng H.B., Guo R.R., Tian Y., Pang Z.Q., Yang W.L. (2017). Red blood cell membrane-camouflaged melanin nanoparticles for enhanced photothermal therapy. Biomaterials.

[59] Castano A.P., Demidova T.N., Hamblin M.R. (2004). Mechanisms in photodynamic therapy: Part one-photosensitizers, photochemistry and cellular localization. Photodiagnosis Photodyn. Ther..

[60] Alexiades-Armenakas M. (2006). Laser-mediated photodynamic therapy. Clin. Dermatol..

[61] Zhang C., Zhao D.X., Fang F., Zhu L., Li W.Y., Wang S., Fan Y.Y., Yang J.N., Liu Y.H., Zhang J.F. (2024). Quintuple Free-Radical Therapy: An Ultralong-Retention FAND for NIR-Involved Multiple Site-Acting Hypoxic Tumor Therapy. Adv. Funct. Mater..

[62] Glorieux C., Liu S.H., Trachootham D., Huang P. (2024). Targeting ROS in cancer: Rationale and strategies. Nat. Rev. Drug Discov..

[63] Silva J.N., Filipe P., Morliere P., Maziere J.C., Freitas J.P., de Castro J.L.C., Santus R. (2006). Photodynamic therapies: Principles and present medical applications. Bio-Med. Mater. Eng..

[64] Allison R.R., Moghissi K. (2013). Photodynamic therapy (PDT): PDT mechanisms. Clin. Endosc..

[65] Lipson R.L., Baldes E.J., Olsen A.M. (1961). The use of a derivative of hematoporhyrin in tumor detection. J. Natl. Cancer Inst..

[66] Lin C.Z., Zhang Y.Y., Zhao Q., Sun P.P., Gao Z., Cui S.C. (2021). Analysis of the short-term effect of photodynamic therapy on primary bronchial lung cancer. Lasers Med. Sci..

[67] Misaki T., Hisazumi H., Miyoshi N. (1984). Photoradiation therapy of bladder tumors. Prog. Clin. Biol. Res..

[68] Zhu T.C., Finlay J.C. (2008). The role of photodynamic therapy (PDT) physics. Med. Phys..

[69] Simelane N.W.N., Kruger C.A., Abrahamse H. (2021). Targeted Nanoparticle Photodynamic Diagnosis and Therapy of Colorectal Cancer. Int. J. Mol. Sci..

[70] Komatsu N., Kosai A., Kuroda M., Hamakubo T., Abe T. (2024). Cetuximab-Toxin Conjugate and NPe6 with Light Enhanced Cytotoxic Effects in Head and Neck Squamous Cell Carcinoma In Vitro. Biomedicines.

[71] Regagnon T., Bugnicourt-Moreira L., Ravaz R., Idlas P., Ramousset L., Kouassi M.C., Theodossiou T., Berg K., Menendez-Miranda M., Gref R. (2023). Photoresponsive liposomes and LipoParticles by incorporating a photosensitizer agent in their lipid membrane. J. Photochem. Photobiol. A Chem..

[72] Chen J.J., Yan M.Q., Huang K.S., Xue J.P. (2023). Novel molecular photosensitizer with simultaneously GSH depletion, aggregation inhibition and accelerated elimination for improved and safe photodynamic therapy. Eur. J. Med. Chem..

[73] Chizenga E.P., Abrahamse H. (2023). Design and assembly of a nanoparticle, antibody, phthalocyanine scaffold for intracellular delivery of photosensitizer to human papillomavirus-transformed cancer cells. Artif. Cells Nanomed. Biotechnol..

[74] Xuan M.J., Shao J.X., Zhao J., Li Q., Dai L.R., Li J.B. (2018). Magnetic mesoporous silica nanoparticles cloaked by red blood cell membranes: Applications in cancer therapy. Angew. Chem. -Int. Ed..

[75] Wang J., Li Y.Y., Nie G.J., Zhao Y.L. (2019). Precise design of nanomedicines: Perspectives for cancer treatment. Natl. Sci. Rev..

[76] Pei Q., Hu X.L., Zheng X.H., Liu S., Li Y.W., Jing X.B., Xie Z.G. (2018). Light-activatable red blood cell membrane-camouflaged dimeric prodrug nanoparticles for synergistic photodynamic/chemotherapy. Acs Nano.

[77] Liu W.L., Liu T., Zou M.Z., Yu W.Y., Li C.X., He Z.Y., Zhang M.K., Liu M.D., Li Z.H., Feng J. (2018). Aggressive man-made red blood cells for hypoxia-resistant photodynamic therapy. Adv. Mater..

[78] Ding L., Wu Y.N., Wu M., Zhao Q.F., Li H.S., Liu J.F., Liu X.L., Zhang X.L., Zeng Y.Y. (2021). Engineered Red Blood Cell Biomimetic Nanovesicle with Oxygen Self-Supply for Near-Infrared-II Fluorescence-Guided Synergetic Chemo-Photodynamic Therapy against Hypoxic Tumors. Acs Appl. Mater. Interfaces.

[79] Xu L.L., Gao F., Fan F., Yang L.H. (2018). Platelet membrane coating coupled with solar irradiation endows a photodynamic nanosystem with both improved antitumor efficacy and undetectable skin damage. Biomaterials.

[80] Mai X.L., Zhang Y.W., Fan H.J., Song W.T., Chang Y., Chen B., Shi J., Xin X.Y., Teng Z.G., Sun J.F. (2020). Integration of immunogenic activation and immunosuppressive reversion using mitochondrial-respiration-inhibited platelet-mimicking nanoparticles. Biomaterials.

[81] Geranpayehvaghei M., Dabirmanesh B., Khaledi M., Atabakhshi-Kashi M., Gao C., Taleb M., Zhang Y.L., Khajeh K., Nie G.J. (2021). Cancer-associated-platelet-inspired nanomedicines for cancer therapy. Wiley Interdiscip. Rev.-Nanomed. Nanobiotechnology.

[82] Klingenberg R., Hansson G.K. (2009). Treating inflammation in atherosclerotic cardiovascular disease: Emerging therapies. Eur. Heart J..

[83] Matthay M.A., Ware L.B., Zimmerman G.A. (2012). The acute respiratory distress syndrome. J. Clin. Investig..

[84] Wong B.W., Meredith A., Lin D., McManus B.M. (2012). The biological role of inflammation in atherosclerosis. Can. J. Cardiol..

[85] Shaul M.E., Fridlender Z.G. (2017). Neutrophils as active regulators of the immune system in the tumor microenvironment. J. Leukoc. Biol..

[86] Grivennikov S.I., Greten F.R., Karin M. (2010). Immunity, inflammation, and cancer. Cell.

[87] Frasco M.F., Almeida G.M., Santos-Silva F., Pereira M.D., Coelho M.A.N. (2015). Transferrin surface-modified PLGA nanoparticles-mediated delivery of a proteasome inhibitor to human pancreatic cancer cells. J. Biomed. Mater. Res. Part A.

[88] Suk J.S., Xu Q.G., Kim N., Hanes J., Ensign L.M. (2016). PEGylation as a strategy for improving nanoparticle-based drug and gene delivery. Adv. Drug Deliv. Rev..

[89] Kang T., Zhu Q.Q., Wei D., Feng J.X., Yao J.H., Jiang T.Z., Song Q.X., Wei X.B., Chen H.Z., Gao X.L. (2017). Nanoparticles coated with neutrophil membranes can effectively treat cancer metastasis. Acs Nano.

[90] Liu J.J., Liu K., Feng L.Z., Liu Z., Xu L.G. (2017). Comparison of nanomedicine-based chemotherapy, photodynamic therapy and photothermal therapy using reduced graphene oxide for the model system. Biomater. Sci..

[91] Gallagher-Colombo S.M., Miller J., Cengel K.A., Putt M.E., Vinogradov S.A., Busch T.M. (2015). Erlotinib Pretreatment Improves Photodynamic Therapy of Non-Small Cell Lung Carcinoma Xenografts via Multiple Mechanisms. Cancer Res..

[92] Yang K., Wan J.M., Zhang S.A., Zhang Y.J., Lee S.T., Liu Z.A. (2011). In vivo pharmacokinetics, long-term biodistribution, and toxicology of PEGylated graphene in mice. Acs Nano.

[93] Sun L.H., Li Q., Hou M.M., Gao Y., Yang R.H., Zhang L., Xu Z.G., Kang Y.J., Xue P. (2018). Light-activatable Chlorin e6 (Ce6)-imbedded erythrocyte membrane vesicles camouflaged Prussian blue nanoparticles for synergistic photothermal and photodynamic therapies of cancer. Biomater. Sci..

[94] Liu J.Y., Smith S., Wang C.Z. (2023). Photothermal attenuation of cancer cell stemness, chemoresistance, and migration using CD44-targeted MoS_2_ nanosheets. Nano Lett..

[95] Zhang T., Liu H., Li L., Guo Z.Y., Song J., Yang X.Y., Wan G.Y., Li R.S., Wang Y.S. (2021). Leukocyte/platelet hybrid membrane-camouflaged dendritic large pore mesoporous silica nanoparticles co-loaded with photo/chemotherapeutic agents for triple negative breast cancer combination treatment. Bioact. Mater..

[96] Khalil D.N., Smith E.L., Brentjens R.J., Wolchok J.D. (2016). The future of cancer treatment: Immunomodulation, CARs and combination immunotherapy. Nat. Rev. Clin. Oncol..

[97] Xu Z.H., Wang Y.H., Zhang L., Huang L. (2014). Nanoparticle-delivered transforming growth factor-beta siRNA enhances vaccination against advanced melanoma by modifying tumor microenvironment. Acs Nano.

[98] Feng B., Niu Z.F., Hou B., Zhou L., Li Y.P., Yu H.J. (2020). Enhancing triple negative breast cancer immunotherapy by ICG-templated self-assembly of paclitaxel nanoparticles. Adv. Funct. Mater..

[99] Liu S., Zhang T.S., Li S.S., Wu Q.Y., Wang K.X., Xu X.C., Lu M.Z., Shao R.G., Zhao W.L., Liu H.Y. (2023). Biomimetic nanobomb for synergistic therapy with inhibition of cancer stem cells. Small.

[100] Fathima J.K., Lavanya V., Jamal S., Ahmed N. (2022). The Effectiveness of Various Chemotherapeutic Agents in Cancer Treatment. Curr. Pharmacol. Rep..

[101] Al Saihati H.A., Rabaan A.A. (2023). Cellular resistance mechanisms in cancer and the new approaches to overcome resistance mechanisms chemotherapy. Saudi Med. J..

[102] Pearce A., Haas M., Viney R., Pearson S.A., Haywood P., Brown C., Ward R. (2017). Incidence and severity of self-reported chemotherapy side effects in routine care: A prospective cohort study. PLoS ONE.

[103] Chen Z.Y., Yue Z.Q., Yang K.Q., Li S.L. (2022). Nanomaterials: Small particles show huge possibilities for cancer immunotherapy. J. Nanobiotechnology.

[104] Zhu Y.X., Jia H.R., Guo Y.X., Liu X.Y., Zhou N.X., Liu P.D., Wu F.G. (2021). Repurposing Erythrocytes as a “Photoactivatable Bomb”: A General Strategy for Site-Specific Drug Release in Blood Vessels. Small.

[105] Yang Z.W., Shi C.S., Cheng D.L., Wang Y., Xing Y., Du F.F., Wu F.F., Jin Y., Dong Y.L., Li M.L. (2022). Biomimetic nanomaterial-facilitated oxygen generation strategies for enhancing tumour treatment outcomes. Front. Bioeng. Biotechnol..

[106] Wang S., Yin Y.P.C., Song W., Zhang Q., Yang Z.J., Dong Z.L., Xu Y., Cai S.J., Wang K., Yang W.L. (2020). Red-blood-cell-membrane-enveloped magnetic nanoclusters as a biomimetic theranostic nanoplatform for bimodal imaging-guided cancer photothermal therapy. J. Mater. Chem. B.

[107] Li J.Q., Zhao R.X., Yang F.M., Qi X.T., Ye P.K., Xie M. (2022). An erythrocyte membrane-camouflaged biomimetic nanoplatform for enhanced chemo-photothermal therapy of breast cancer. J. Mater. Chem. B.

[108] Huang S.N., Song C.Z., Miao J.X., Zhu X.L., Jia Y.Y., Liu Y.F., Fu D.J., Li B.Y., Miao M.S., Duan S.F. (2023). Red blood cell membrane-coated functionalized Au nanocage as a biomimetic platform for improved MicroRNA delivery in hepatocellular carcinoma. Int. J. Pharm..

[109] Wang W.B., Cheng Z.Y., Xing H., Zhou S.H., Ye Q.Z., Xiong G.F., Wang G.H., Ma D. (2024). Red cell membrane-coating Prussian blue for combined photothermal and NO gas therapy for nasopharyngeal carcinoma. J. Mater. Chem. B.

[110] Gao J.B., Wang F., Wang S.H., Liu L., Liu K., Ye Y.C., Wang Z., Wang H., Chen B., Jiang J.M. (2020). Hyperthermia-triggered on-demand biomimetic nanocarriers for synergetic photothermal and chemotherapy. Adv. Sci..

[111] Yang J.X., Teng Y.L., Fu Y., Zhang C.Y. (2019). Chlorins e6 loaded silica nanoparticles coated with gastric cancer cell membrane for tumor specific photodynamic therapy of gastric cancer. Int. J. Nanomed..

[112] Zhang D., Ye Z.J., Wei L., Luo H.B., Xiao L.H. (2019). Cell membrane-coated porphyrin metal-organic frameworks for cancer cell targeting and O_2_-evolving photodynamic therapy. Acs Appl. Mater. Interfaces.

[113] Sun H.P., Su J.H., Meng Q.S., Yin Q., Chen L.L., Gu W.W., Zhang Z.W., Yu H.J., Zhang P.C., Wang S.L. (2017). Cancer cell membrane-coated gold nanocages with hyperthermia-triggered drug release and homotypic target inhibit growth and metastasis of breast cancer. Adv. Funct. Mater..

[114] Fang R.H., Hu C.M.J., Luk B.T., Gao W.W., Copp J.A., Tai Y.Y., O’Connor D.E., Zhang L.F. (2014). Cancer cell membrane-coated nanoparticles for anticancer vaccination and drug delivery. Nano Lett..

[115] Li M.H., Cui X.Y., Wei F., Wang Z., Han X.J. (2021). Red blood cell membrane-coated biomimetic upconversion nanoarchitectures for synergistic chemo-photodynamic therapy. New J. Chem..

[116] Zhang N., Ping W., Rao K.X., Zhang Z.L., Huang R., Zhu D.M., Li G.X., Ning S.P. (2024). Biomimetic copper-doped polypyrrole nanoparticles induce glutamine metabolism inhibition to enhance breast cancer cuproptosis and immunotherapy. J. Control. Release.

[117] Duo Y.H., Quan L., Zhu D.M., Bin Z., Luo G.H., Wang F.B., Chen J.H., Cao Y.H. (2022). Proof of concept for dual anticancer effects by a novel nanomaterial-mediated cancer cell killing and nano-radiosensitization. Chem. Eng. J..

[118] Li Q., Su R., Bao X., Cao K.X., Du Y.Y., Wang N.Y., Wang J.F., Xing F., Yan F., Huang K.K. (2022). Glycyrrhetinic acid nanoparticles combined with ferrotherapy for improved cancer immunotherapy. Acta Biomater..

[119] Wu J.H., Ma T., Zhu M.N., Mu J.F., Huang T.C., Xu D.H., Lin N.M., Gao J.Q. (2024). A Pluripotential Neutrophil-Mimic Nanovehicle Modulates Immune Microenvironment with Targeted Drug Delivery for Augmented Antitumor Chemotherapy. Acs Nano.

[120] Liu X.Z., Wen Z.J., Li Y.M., Sun W.R., Hu X.Q., Zhu J.Z., Li X.Y., Wang P.Y., Pedraz J.L., Lee J.H. (2023). Bioengineered Bacterial Membrane Vesicles with Multifunctional Nanoparticles as a Versatile Platform for Cancer Immunotherapy. Acs Appl. Mater. Interfaces.

[121] Huang X., Wang L.T., Guo H.Y., Zhang W.Y. (2023). Macrophage membrane-coated nanovesicles for dual-targeted drug delivery to inhibit tumor and induce macrophage polarization. Bioact. Mater..

[122] Guo Q.Y., Wang S.M., Xu R.B., Tang Y.N., Xia X.H. (2024). Cancer cell membrane-coated nanoparticles: A promising anti-tumor bionic platform. Rsc Adv..

